# Chemotherapy-Induced Thrombotic Microangiopathy in a Pediatric Patient With a Single Functioning Kidney: A Case Report and Review of the Literature

**DOI:** 10.7759/cureus.105034

**Published:** 2026-03-11

**Authors:** Maryam Jamil, Bilal Shahzad Azam Khan, Aqeela Rashid, Najma Shaheen

**Affiliations:** 1 Pediatric Oncology, Shaukat Khanum Memorial Cancer Hospital and Research Centre, Lahore, PAK; 2 Nephrology, Shaukat Khanum Memorial Cancer Hospital and Research Centre, Lahore, PAK

**Keywords:** metastatic disease, radiation nephritis, renal insufficiency, thrombotic microangiopathy, wilm’s tumor

## Abstract

Thrombotic microangiopathy (TMA) is a rare but life-threatening complication in patients with cancer and can be difficult to distinguish from other treatment-related toxicities. We report the case of an eight-year-old girl with metastatic Wilms’ tumor and a solitary kidney after left nephrectomy who developed biopsy-confirmed TMA following intensive multimodal therapy. She received multiple lines of chemotherapy, including actinomycin D, vincristine, cyclophosphamide, doxorubicin, and carboplatin, as well as radiotherapy to the bilateral lungs, nephrectomy bed, and whole abdomen. Several months into treatment, she developed progressive renal insufficiency with rising serum creatinine, microscopic hematuria, proteinuria, and subsequent extra-renal manifestations, including heart failure and new-onset seizures. Laboratory evaluation showed acute kidney injury, and renal biopsy demonstrated focal fibrin thrombi with mesangiolysis consistent with acute TMA, alongside mild acute tubular injury and non-specific immunofluorescence findings. In the absence of access to ADAMTS13 and complement genetic testing, chemotherapy-induced TMA, most likely related to cumulative exposure to carboplatin or doxorubicin in the context of prior radiotherapy, was considered the leading diagnosis. Withdrawal of chemotherapy was followed by partial renal recovery. This case highlights the need for a high index of suspicion for drug-induced TMA in pediatric oncology patients who develop new renal dysfunction and neurological or cardiac symptoms during or after exposure to multiple potentially endothelial-toxic agents. It also highlights the central role of renal biopsy in diagnosis and the importance of early recognition and treatment modification to preserve renal function, particularly in children with limited nephron reserve.

## Introduction

Thrombotic microangiopathy (TMA) is a rare but life-threatening condition that typically presents with the classic triad of microangiopathic hemolytic anemia, thrombocytopenia, and signs of ischemic organ damage, most often involving the kidneys and the central nervous system [1,2]. TMA results from endothelial injury and microvascular thrombosis, which cause red blood cell destruction and organ dysfunction [3]. Diagnosing TMA in patients with cancer is challenging because its clinical features can mimic complications of the underlying malignancy, such as tumor-related coagulopathy, infection, or chemotherapy-related toxicity [4].

Patients with cancer can develop three main types of TMA: paraneoplastic TMA, which is directly induced by the malignancy; TMA related to complications after hematopoietic stem cell transplantation; and drug-induced TMA (DI-TMA) [3]. DI-TMA has been increasingly recognized as an important cause of secondary TMA and presents a significant clinical challenge because it can interrupt or delay life-saving cancer therapies [1,2]. Two principal mechanisms of DI-TMA have been described: immune-mediated, drug-dependent antibodies that rapidly injure endothelial cells, and dose-dependent or toxic DI-TMA, which worsens over time with repeated exposure to agents that directly damage endothelial cells [2,5].

Several chemotherapeutic agents, including platinum-based drugs (carboplatin and cisplatin), gemcitabine, and anthracyclines (doxorubicin), have been implicated in DI-TMA. The incidence of DI-TMA varies by drug and is influenced by patient characteristics such as age and sex [1,2,4]. Patients with underlying comorbidities or concomitant therapies, such as radiotherapy, may have an increased risk of TMA because of the additional endothelial injury [4]. This risk is particularly challenging in pediatric patients with cancer, who may have unique health vulnerabilities, such as a solitary kidney, while clinicians balance the competing risks of cancer progression and treatment-related toxicity.

This report describes a child with metastatic Wilms’ tumor who developed biopsy-confirmed TMA after chemotherapy and radiotherapy. The working diagnosis was non-immune, chemotherapy-induced TMA in the setting of radiation nephritis. This case aims to add to the limited literature on refractory secondary TMA in pediatric oncology and to highlight current treatment strategies. The case was presented as a poster at the 24th Shaukat Khanum Cancer Symposium held from October 24 to 26, 2025.

## Case presentation

**Figure 1:**
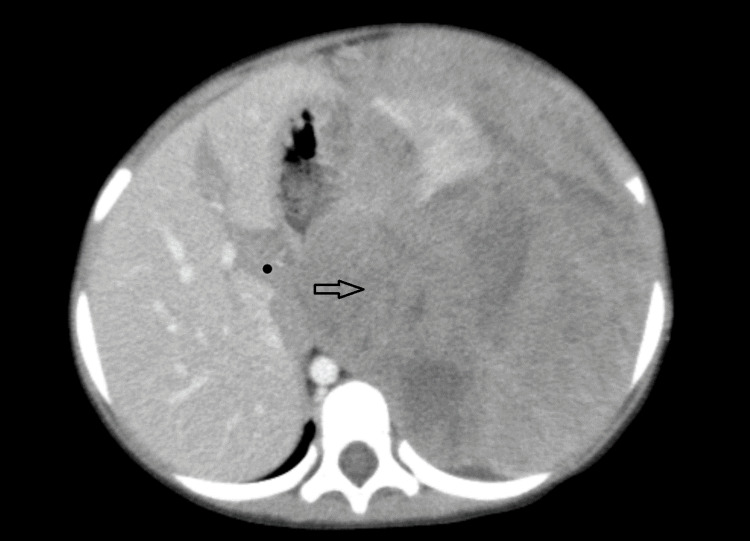
Axial CT image (19-02-2024) showing a left hemiabdomen mass. CT, computed tomography.

**Figure 2:**
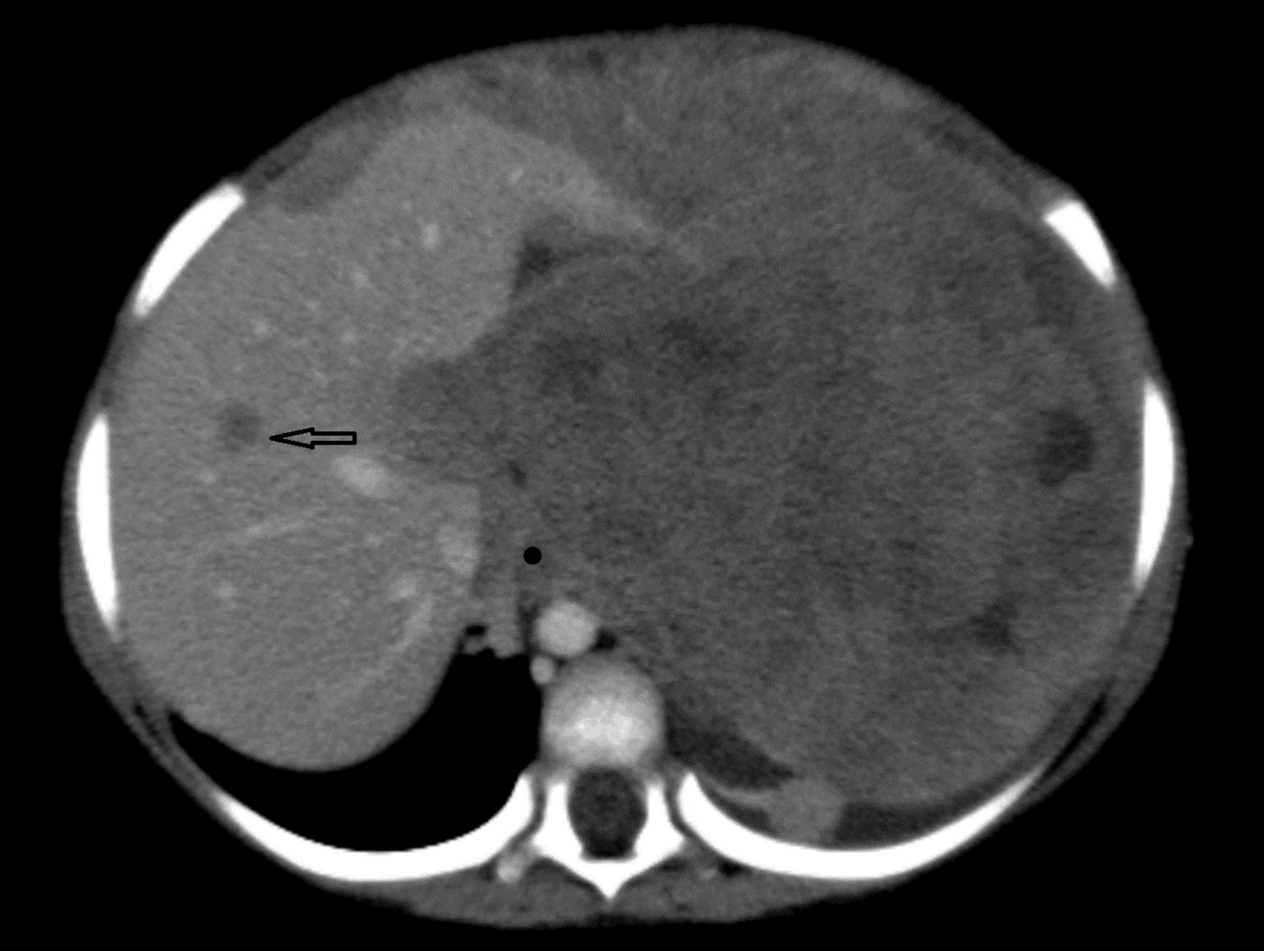
CT image (19-02-2024) showing a hepatic lesion. CT, computed tomography.

**Figure 3:**
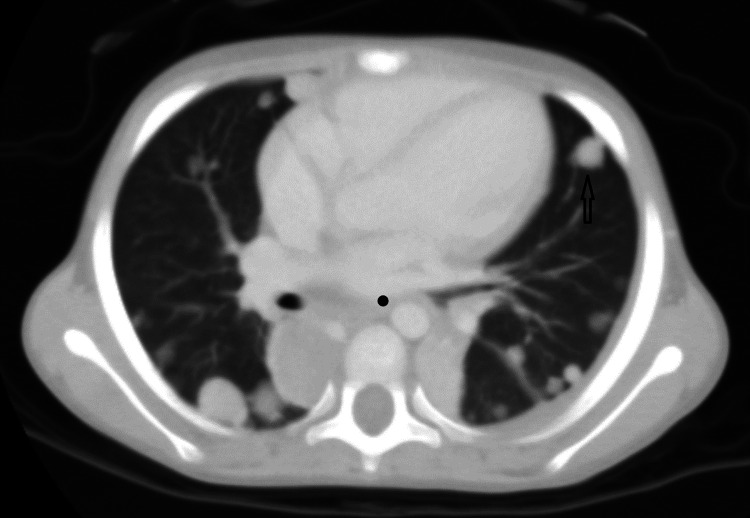
CT axial lung window (19-02-2024) showing multiple pulmonary metastatic deposits. CT, computed tomography.

**Figure 4:**
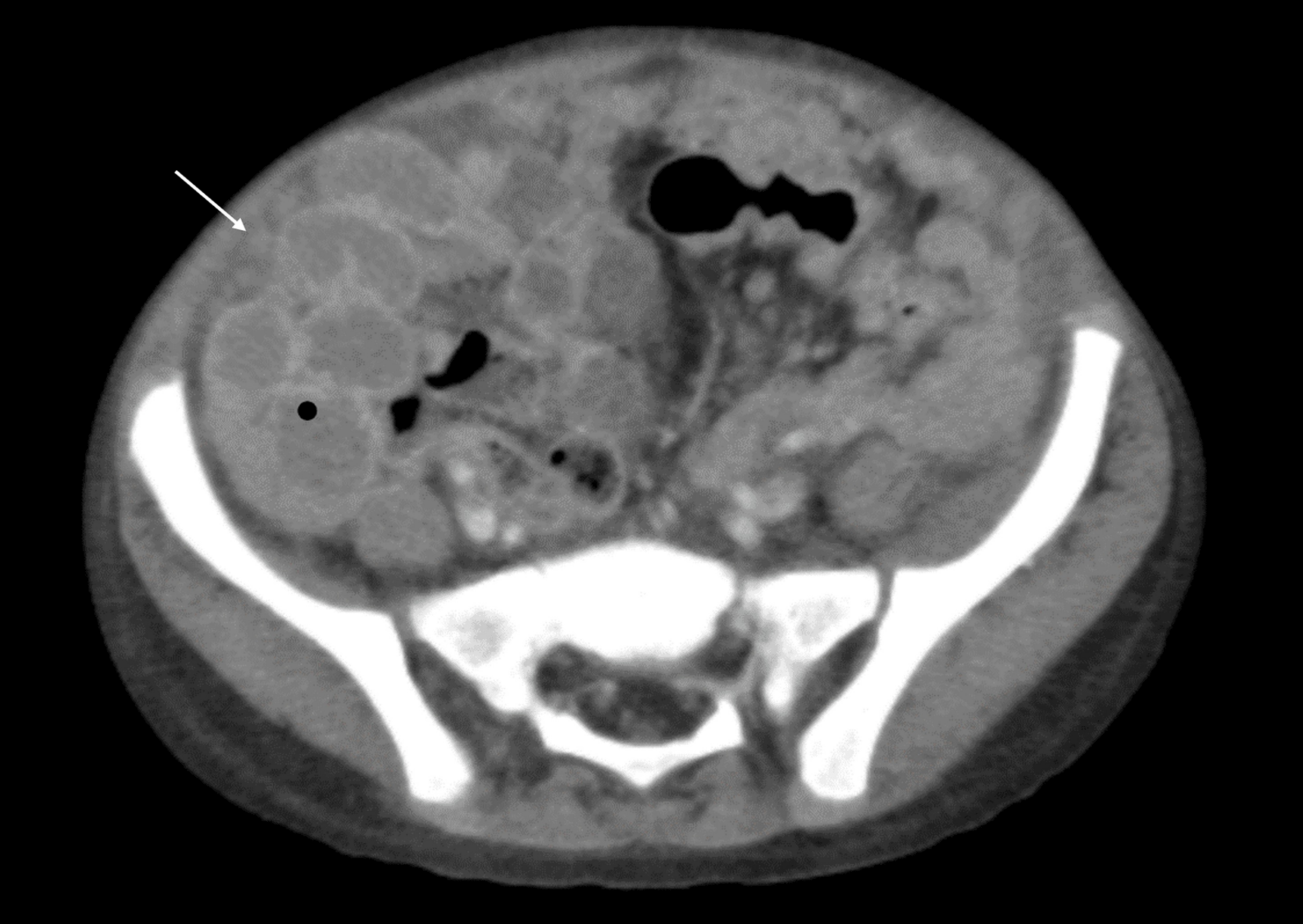
CT image (19-02-2024) showing peritoneal nodules. CT, computed tomography.

**Figure 5:**
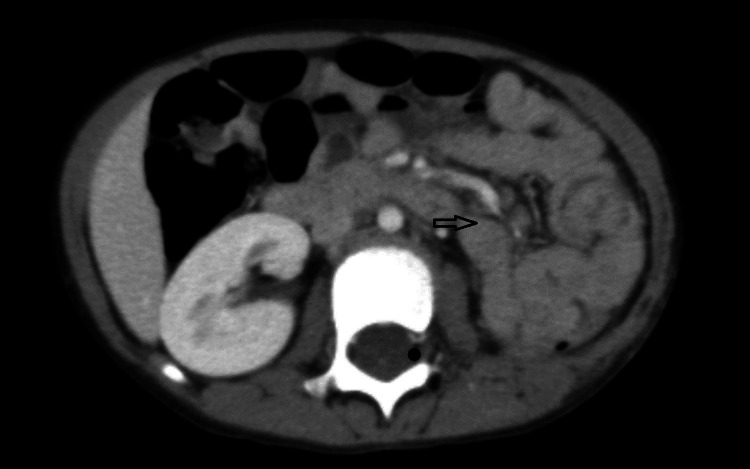
Axial CT image (21-08-2024) showing satisfactory postsurgical changes of left nephrectomy with no evidence of local recurrence. CT, computed tomography.

An eight-year-old girl presented with gross hematuria, reduced appetite, and abdominal pain. Clinicians identified a left renal mass, and she underwent a left-sided nephrectomy in Peshawar, Pakistan, in January 2024. Histopathological examination confirmed Wilms’ tumor. By the time she presented to our institution in February 2024, she had recurrent disease with a large left renal bed mass and widespread metastases involving the lungs, liver, peritoneum, muscles, and skin (Figures 1-4). She received six cycles of adjuvant chemotherapy with actinomycin D, doxorubicin, and vincristine between February and April 2024. An interim computed tomography scan showed a good response to chemotherapy, with resolution of liver lesions, near-complete resolution of lung lesions, and significant regression of the nephrectomy bed mass (Figure 5). She completed radiotherapy to the bilateral lungs, left nephrectomy bed, and whole abdomen on October 23, 2024. The right kidney received a mean dose of 20 Gy delivered in 1.5-Gy fractions. From May 2024 to January 2025, she received additional chemotherapy consisting of four cycles of cyclophosphamide plus doxorubicin alternating with five cycles of carboplatin plus etoposide. Her last doses of carboplatin and doxorubicin were administered on November 23, 2024, and December 31, 2024, respectively. During this period, she had multiple emergency department visits and hospital admissions for febrile neutropenia and severe thrombocytopenia.

**Figure 6:**
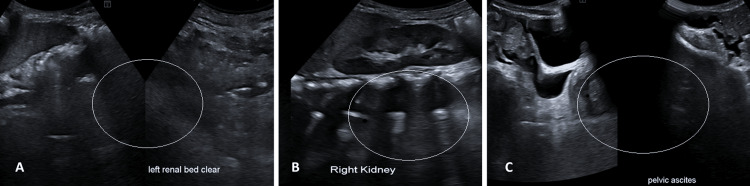
Ultrasonography (25-03-2025) showing no sonographic evidence of disease recurrence in the left nephrectomy bed (A), with a normal right kidney (B) and mild pelvic ascites (C).

Her baseline serum creatinine was 0.3 mg/dL on November 21, 2024, but progressively increased to 1.3 mg/dL by February 9, 2025. Imaging showed mild ascites without evidence of disease recurrence (Figure 6). Urinalysis demonstrated microscopic hematuria and proteinuria, and renal ultrasonography showed no structural abnormalities. Additional laboratory findings included a urine protein-to-creatinine ratio of 3.975 g/g, a platelet count of 63 × 10³/µL, a hemoglobin of 8 g/dL, a serum albumin of 3.6 g/dL, and a positive antinuclear antibody (ANA) test at a low titer (1:160). Complement levels (C3 and C4) were within normal limits.

The patient was referred to nephrology. History and physical examination were negative for signs or symptoms suggestive of systemic lupus erythematosus or thrombosis. Her blood pressure was normal at the time of diagnosis, and she had no edema. Given the progressive renal impairment and positive ANA result, clinicians initiated oral prednisolone and planned a renal biopsy.

By mid-March 2025, the patient developed early signs of heart failure. Transthoracic echocardiography showed left ventricular dilation, a pericardial effusion, and a reduced ejection fraction. Clinicians initiated captopril and diuretic therapy. She was also admitted with new-onset seizures. Brain magnetic resonance imaging and lumbar puncture findings were unremarkable. Levetiracetam was started for seizure prophylaxis, and close follow-up with pediatric oncology and nephrology was arranged.

**Figure 7:**
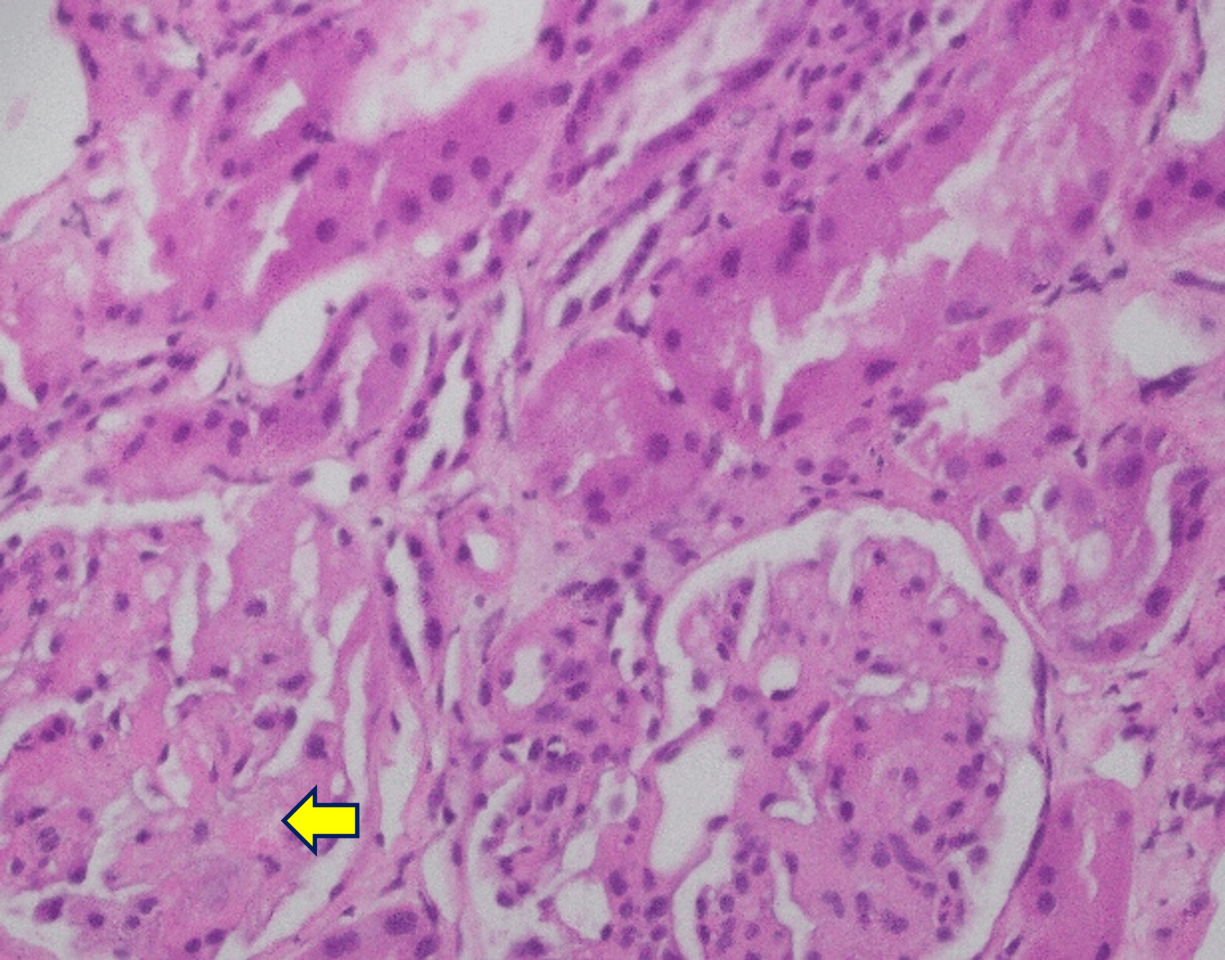
Hematoxylin and eosin stain, light microscopy, 40× All glomeruli show features of acute thrombotic microangiopathy, characterized by focal fibrin thrombi in the peripheral capillary loops (arrow) with mesangiolysis.

**Figure 8:**
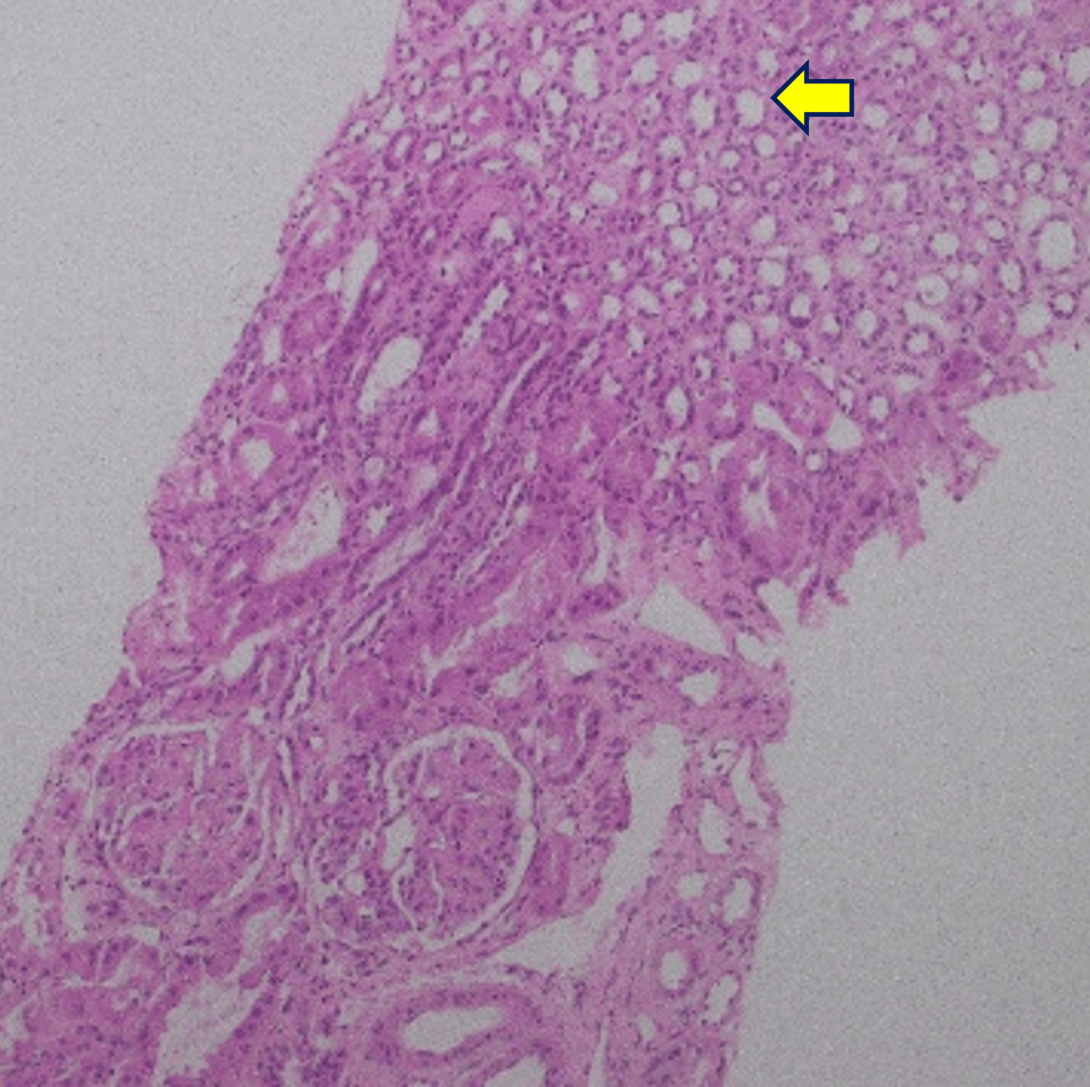
Hematoxylin and eosin stain, light microscopy, 10× Mild acute tubular injury is present, with dilated tubules showing loss of brush borders and hyperchromatic tubular epithelial nuclei (arrow).

**Figure 9:**
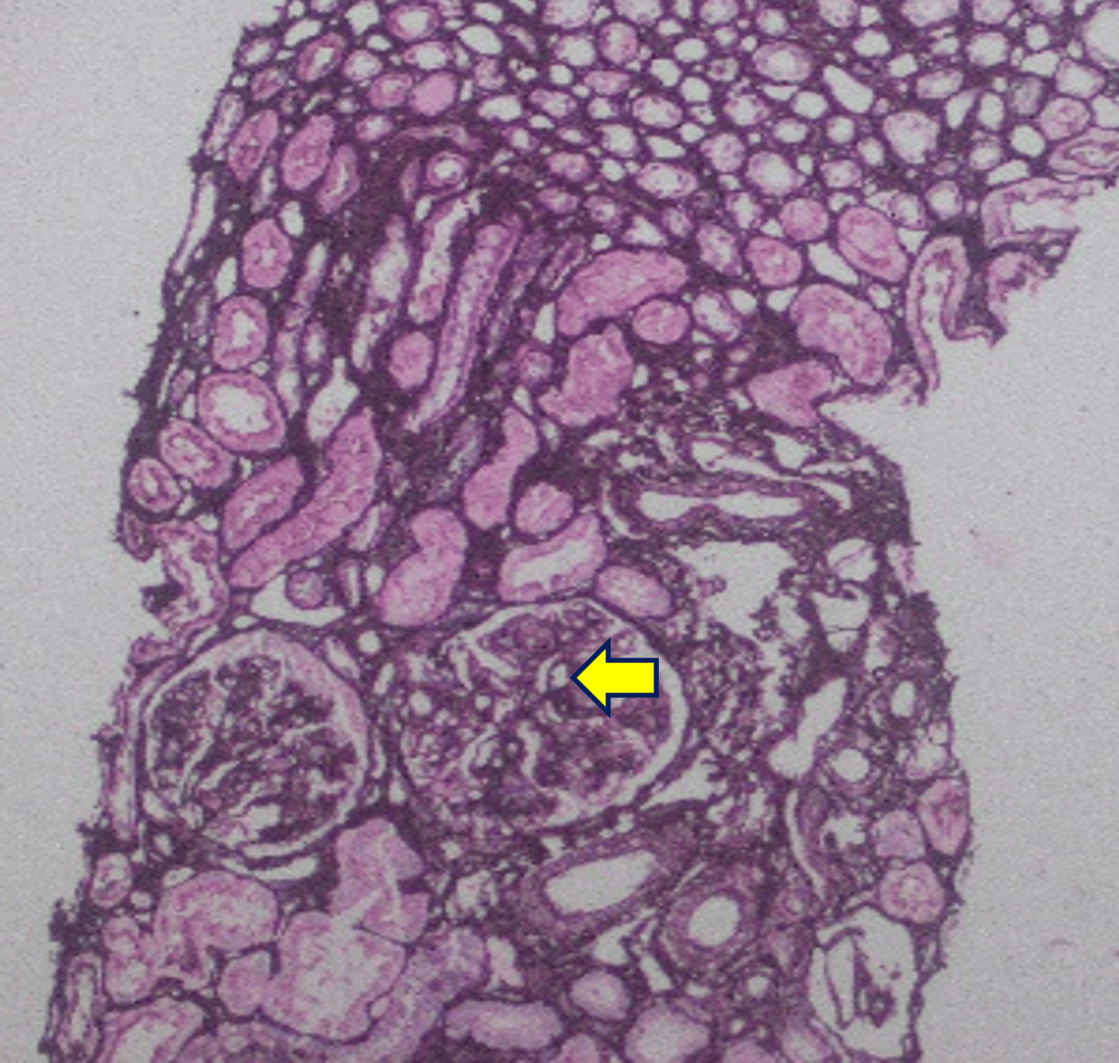
Jones methenamine silver stain, light microscopy, 10× Glomeruli demonstrate prominent endothelial cells (arrow). No proliferative changes are observed, and glomerular basement membranes do not show double contouring.

**Figure 10:**
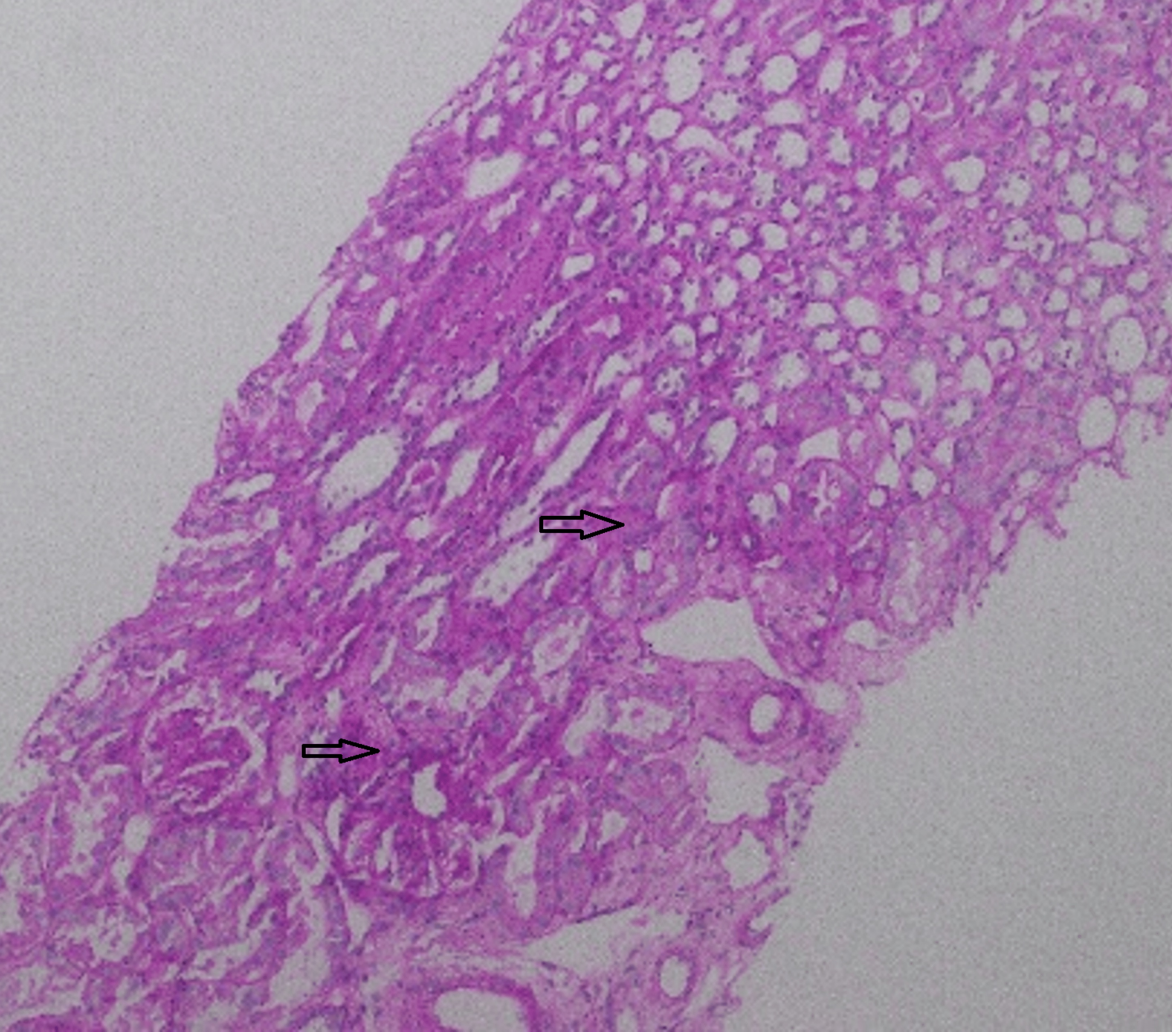
Periodic acid-Schiff stain, light microscopy, 10×, of the renal biopsy specimen Arrows indicate dilated renal tubules with attenuated epithelial lining and mild acute tubular injury.

**Figure 11:**
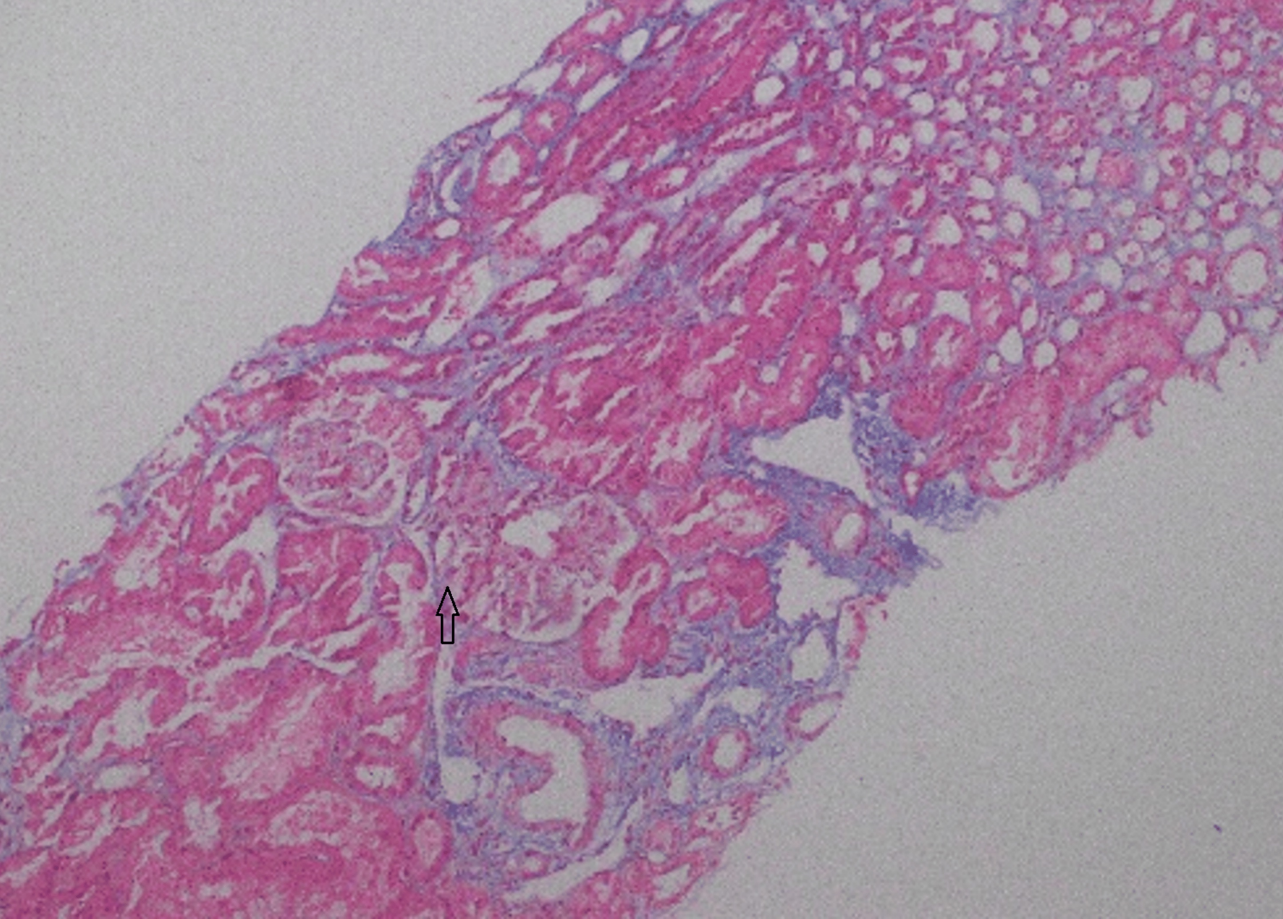
Masson trichrome stain, light microscopy, 10×, of the renal biopsy specimen The stain demonstrates mild interstitial expansion without significant fibrosis, with overall preservation of renal architecture.

Renal biopsy demonstrated focal fibrin thrombi in the peripheral capillary loops with mesangiolysis on light microscopy, findings consistent with acute TMA (Figures 7-11). Mild acute tubular injury was also present. Immunofluorescence was largely negative, apart from immunoglobulin M staining in sclerotic areas and moderate granular membranous staining for complement component 1q (C1q). The patient’s condition did not improve with corticosteroids; therefore, corticosteroids were discontinued.

Unfortunately, no hemolysis workup was performed at the time because TMA was not initially suspected. Total bilirubin, obtained as part of liver function testing, was within normal limits. The patient received cumulative doses of 190.5 mg doxorubicin (300 mg/m²) and 1767 mg carboplatin (2850 mg/m²). Clinicians considered chemotherapy-induced TMA the most likely diagnosis, and further chemotherapy was discontinued. Plasmapheresis and eculizumab were not pursued because the clinical picture was not consistent with thrombotic thrombocytopenic purpura (TTP) or complement-mediated TMA.

After discontinuing chemotherapy, serum creatinine improved to 0.8 mg/dL in April 2025. Using the modified Schwartz equation, this corresponds to an estimated glomerular filtration rate of approximately 55 mL/min/1.73 m², consistent with chronic kidney disease stage IIIa. She did not require renal replacement therapy because renal function improved after chemotherapy cessation, and there were no acute indications for dialysis. At the time of writing, the patient remains in clinical remission with regard to her malignancy. A detailed treatment protocol, delivered doses, and cumulative exposures are summarized in Table 1.

**Table 1:** Chemotherapy protocols administered and cumulative doses received CAR, carboplatin; CYC, cyclophosphamide; DOX, doxorubicin; ET, etoposide; HRM, high-risk metastatic; PREOP, preoperative; VA, vincristine + dactinomycin; VAD, vincristine + dactinomycin + doxorubicin.

Protocol	Cycles, N	Chemotherapy days	Drug	Standard dose	Doses administered, n	Doses skipped, n	Total dose administered (mg)
PREOP VAD	1	Weeks 1-6	Vincristine	1.5 mg/m^2^	6	0	5.88
PREOP VAD	1	Weeks 1, 3, and 5	Dactinomycin	0.045 mg/kg	2	1 (drug unavailable)	1.36
PREOP VAD	1	Weeks 1 and 5	Doxorubicin	50 mg/m^2^	2	0	65
PREOP VA	1	Days 1, 8, 15, and 22	Vincristine	1.5 mg/m^2^	4	0	3.72
PREOP VA	1	Days 1 and 15	Dactinomycin	0.045 mg/kg	2	0	1.26
HRM CYC/DOX	4	Days 1-3	Cyclophosphamide	450 mg/m^2^	12	0	3388.5
HRM CYC/DOX	4	Day 1	Doxorubicin	50 mg/m^2^	4	0	125.5
HRM CAR/ET	4	Days 1-3	Carboplatin	200 mg/m^2^	12	0	1488
HRM CAR/ET	4	Days 1-3	Etoposide	150 mg/m^2^	12	0	1116
HRM CAR/ET	1	Days 1-3	Carboplatin	150 mg/m^2^	3	0	279
HRM CAR/ET	1	Days 1-3	Etoposide	112.5 mg/m^2^	3	0	209.2

## Discussion

DI-TMA is a form of TMA that occurs after exposure to a specific drug. Numerous drugs are associated with the development of TMA, including chemotherapeutic agents, immunosuppressants, opioids, and monoclonal antibodies, many of which are used in oncology. The pathophysiology is broadly divided into immune-mediated and non-immune-mediated mechanisms. Immune-mediated DI-TMA develops when drug-dependent antibodies form against a particular agent [6]. In the presence of the drug, these antibodies damage endothelial cells, leading to severe symptoms that develop rapidly after exposure. In non-immune DI-TMA, symptoms develop more gradually after repeated exposure and occur in a dose-dependent manner.

In our case, the patient received several chemotherapeutic agents known to be associated with DI-TMA, including vincristine, carboplatin, and doxorubicin. Because her last dose of vincristine was in April 2024, this drug is less likely to be the cause of her TMA. Kidney function began to deteriorate throughout December 2024, with serum creatinine peaking in early February 2025, a timeline that implicates doxorubicin and/or carboplatin as potential contributors. Additionally, she had received previous cycles of these agents without kidney injury, hematuria, or proteinuria until December 2024, which might suggest a possible non-immune, cumulative toxic mechanism. Based on our literature review, platinum-based chemotherapy with cisplatin is frequently reported to be strongly associated with TMA, whereas carboplatin, an analog of cisplatin, has comparatively few documented case reports [7]. There are also case reports of TMA associated with doxorubicin [8]. It is also notable that the patient developed cardiac dysfunction and seizures, which may reflect organ ischemia related to DI-TMA; however, doxorubicin-induced cardiomyopathy should also be considered.

When evaluating suspected DI-TMA in a patient receiving multiple chemotherapy agents, it is important to rule out other causes, including TTP, complement-mediated TMA (formerly known as atypical hemolytic uremic syndrome [HUS]) due to dysregulation of the alternative complement pathway, and Shiga toxin-producing *Escherichia coli*-associated HUS [9]. Evaluation should include a negative Coombs test, low haptoglobin, elevated lactate dehydrogenase, and a peripheral blood smear to identify schistocytes, as well as documentation that ADAMTS13 activity is not severely reduced. Renal biopsy can often provide diagnostic confirmation and help distinguish TMA from other causes of AKI by showing the characteristic microvascular changes of the condition [10]. In this case, the renal biopsy showed findings consistent with TMA.

Immunofluorescence demonstrated moderate C1q deposition; however, in the absence of clinical features of systemic lupus erythematosus or antiphospholipid antibody syndrome and without broader immune-complex/complement deposition in the glomeruli, this finding most likely represents nonspecific staining, which may occur in association with fibrin microthrombi. Another entity associated with prominent C1q staining is C1q nephropathy, which typically presents with nephrotic syndrome and biopsy findings of minimal change disease, focal segmental glomerulosclerosis, or a proliferative glomerulonephritis pattern [11]. These patterns do not match this patient’s histopathologic findings or clinical presentation. Although TTP and complement-mediated TMA (potentially triggered by stressors such as radiation or infection) were considered, the patient’s improvement in kidney function after discontinuation of chemotherapy alone argues against these diagnoses. Genetic testing for complement pathway mutations may be considered when complement-mediated TMA remains a concern [12]. At our institution, ADAMTS13 testing and genetic testing for complement pathway mutations were not available.

Radiation nephritis can also present with progressive renal insufficiency. In this case, the patient received radiotherapy approximately three months before the onset of kidney dysfunction. Radiation nephritis is a severe complication of renal exposure to ionizing radiation and is characterized by endothelial cell injury. The radiosensitivity of the renal microvasculature is central to the pathophysiology of radiation-induced kidney damage. Ionizing radiation directly injures glomerular and endothelial cells and triggers inflammation, oxidative stress, and the release of prothrombotic factors. Ultimately, these processes impair renal blood flow and function and can produce pathological features that overlap with TMA, including glomerular basement membrane thickening, fibrin tactoids, and microthrombi formation [13]. Careful radiation planning and dose limitation in the pediatric population are essential because pediatric kidneys are more vulnerable to radiation damage than adult kidneys [14]. However, radiation nephritis typically follows an insidious course; proteinuria, new-onset hypertension, and declining renal function often appear months to years after exposure, making it difficult to link symptoms directly to prior treatment [10]. In our patient, the timing and pattern of renal function loss did not fully match this typical course, although radiation nephritis could not be completely excluded.

Because the initial endothelial injury is largely irreversible, treating TMA in a patient with a solitary kidney is challenging. Loss of nephron reserve after nephrectomy places such patients at higher risk of developing chronic kidney disease or end-stage renal disease [15]. The main treatment goals are to manage complications and prevent further glomerular injury due to hyperfiltration. Strict blood pressure control is crucial and often requires antihypertensive agents, especially angiotensin-converting enzyme inhibitors or angiotensin II receptor blockers, although clinicians should use these drugs with caution in patients with rapidly declining glomerular filtration rate [16]. Close monitoring and management of electrolyte and fluid balance are also essential. Although plasma exchange is effective in TTP, its benefit in DI-TMA has not been established, likely because of differences in pathophysiology. A trial of eculizumab, a monoclonal antibody that inhibits the terminal complement pathway, may be considered when complement-mediated TMA is suspected or cannot be definitively ruled out [17].

## Conclusions

This case highlights the diagnostic and therapeutic challenges of DI-TMA in a child with metastatic Wilms’ tumor, prior nephrectomy, and recent radiotherapy. Clinicians should maintain a high index of suspicion for DI-TMA when new-onset AKI, proteinuria, and extra-renal manifestations such as cardiac dysfunction or seizures develop in pediatric oncology patients receiving multiple potentially causative agents. Renal biopsy can be crucial in confirming TMA and distinguishing it from other causes of kidney injury, particularly when laboratory resources to assess ADAMTS13 activity or complement pathway mutations are limited. In patients with a solitary kidney, early recognition of DI-TMA and timely withdrawal of the offending chemotherapeutic agents may allow partial renal recovery and help preserve remaining nephron function. Careful chemotherapy selection, vigilant monitoring for endothelial toxicity, and judicious radiation planning are essential to balance oncologic control with the risk of irreversible renal damage in this vulnerable population.
